# Beyond Cost Centers: The True Economic Contribution of Nursing in Healthcare

**DOI:** 10.1177/23779608251356144

**Published:** 2025-06-30

**Authors:** Ahmad A. Abujaber, Abdulqadir J. Nashwan

**Affiliations:** Nursing Department, Hamad Medical Corporation, Doha, Qatar; Nursing & Midwifery Research Department, Hamad Medical Corporation, Doha, Qatar

*Dear Editor*,

The economic value of nursing is defined as “the return on investment (ROI) in nursing human capital, reflected in improved patient, organizational, and workforce outcomes” (Yakusheva et al., 2024). Nurses form the backbone of healthcare systems, delivering care that enhances patient outcomes, operational efficiency, and cost containment. Despite these critical contributions, nursing remains undervalued by executives and policymakers who prioritize revenue generation and cost control (Adams & Nelson, 2009). This undervaluation is rooted in historical biases, insufficient economic evidence, and limited nursing influence in strategic and policy domains (Gunn et al., 2019).

Nursing's undervaluation is historically tied to its origins as a caregiving role predominantly associated with women. Traditionally, nursing was perceived as an extension of domestic responsibilities, leading to its undervaluation in technical expertise and formal education (Clayton-Hathway et al., 2020). Emotional labor, central to nursing, has been overlooked because it is less visible and difficult to quantify, reinforcing marginalization in financial and strategic decisions. This bias persists in classifying nursing services as operational costs rather than as drivers of clinical and economic value (Rutherford, 2012).

To challenge these entrenched stereotypes, nursing must be reframed as a knowledge-based, skill-driven profession integral to healthcare delivery. Demonstrating measurable impacts such as reduced hospital stays, improved patient outcomes, and cost optimization can strengthen this narrative. Amplifying the presence of nursing leaders in strategic discussions is critical for dismantling biases and ensuring the profession's contributions are recognized and valued (Mason et al., 2020). Although nursing's impact on healthcare outcomes, such as reducing readmissions, preventing medical errors, and enhancing efficiency, is well-documented, its role is often underrecognized in strategic healthcare decision making (Aiken et al., 2011). The absence of standardized economic frameworks to quantify these contributions undermines their financial narrative (Adams & Nelson, 2009). Nursing research often focuses on clinical and theoretical metrics like patient satisfaction and quality of care, aligning with the profession's caregiving ethos (Chen et al., 2022). However, these measures must resonate more with executives and policymakers prioritizing financial performance (Bayram et al., 2022; Needleman et al., 2002). Evidence linking nursing interventions to improved outcomes, such as reduced hospital-acquired infections or shorter hospital stays, often fails to translate into monetary terms. This lack of integration weakens nursing's visibility in decision-making processes and financial discussions (Dall et al., 2009; Htay & Whitehead, 2021; Papathanasiou et al., 2024; Stone et al., 2002).

Nursing organizations and academic institutions must consolidate research on nursing's clinical impacts into measurable economic outcomes (Beal, 2012; Gilliss et al., 2021; Pintz et al., 2022). Standardized financial metrics are needed to translate clinical results, such as reduced hospital-acquired infections, into cost savings (Phillips et al., 2019; Stone et al., 2002). A collaborative model could include joint research centers, co-funded projects, or academic-practice partnerships where nurses, educators, and health economists work together. For example, the Academic-Practice Partnership model, as outlined by the American Association of Colleges of Nursing (AACN) and the American Organization for Nursing Leadership (AONL), emphasizes formal relationships, mutual goals, and shared knowledge to advance nursing practice and improve public health (American Association of Colleges of Nursing, 2020). To maximize the potential of this model, healthcare organizations and academic institutions should establish joint strategic planning committees, invest in shared faculty appointments, and implement collaborative quality improvement initiatives. These efforts can help align educational preparation with clinical needs, foster innovation in care delivery, and ensure a robust pipeline of practice-ready nursing professionals. Healthcare leadership requires an understanding of power dynamics and influence (Clarke et al., 2021). However, nursing leadership has traditionally focused on clinical expertise and operational management, with limited engagement in political advocacy and the exercise of authentic and meaningful transformational leadership necessary to influence health policy and systems-level change (Cervera-Gasch et al., 2022). Addressing this gap involves fostering political and economic leadership skills, enabling nurses to advocate effectively for systemic changes (Lunardi et al., 2006; Mena Tudela & González Chordá, 2018; Shariff, 2014)*.* Incorporating healthcare economics and business acumen into nursing curricula equips future leaders to articulate nursing's financial value and navigate strategic decision-making processes (Jennings et al., 2008; Platt et al., 2016; Raftery et al., 2022). Thus, nursing education programs are encouraged to magnify this emphasis within undergraduate and graduate curricula, fostering a generation of nurse leaders proficient in clinical care, financial stewardship, and systems-level thinking (Flaubert et al., 2021).

We must develop robust models that link nursing interventions to tangible financial outcomes, such as cost savings from reduced hospital readmissions or improved staffing levels, to strengthen the case for investing in nursing resources. Nursing leaders need to be equipped with training in healthcare economics and politics to articulate the value of nursing in strategic discussions and influence decisions on resource allocation. Nursing organizations should collaborate with other healthcare stakeholders—such as hospital administrators, physician associations, public health agencies, policymakers, and payer organizations—to advocate for policies recognizing nursing's economic value. Unified efforts can drive changes like equitable resource allocation and the inclusion of nursing metrics in value-based care models.

Case studies that quantify nursing's financial contributions illustrate its strategic value. For example, a nurse-led initiative that reduced central line-associated bloodstream infections (CLABSI) by 40% at an academic medical center resulted in annual savings of $2 million (Stone et al., 2002). Similarly, professional development programs at one hospital led to a 30% reduction in nursing turnover, saving approximately 5% of the organization's operating budget (Waldman et al., 2010). In Italy, community nurse-led education for heart failure patients incurred an additional €1.3 million in costs but generated 247 additional quality-adjusted life years (QALYs), demonstrating cost-effectiveness (Iovino et al., 2024). At an Australian hospital, nurse practitioners helped reduce emergency department waiting times by 40%, yielding $750,000 in additional annual revenue (Jennings et al., 2008). Additionally, a nurse-led program in a Canadian facility achieved a 60% reduction in pressure injuries, resulting in $800,000 in yearly cost savings (Vanderwee et al., 2007). These exemplars collectively underscore the economic and clinical impact of nurse-led innovations.

Despite its measurable economic benefits, nursing care remains undervalued due to historical biases, challenges in quantifying outcomes, and limited advocacy. Nursing organizations and academic institutions must consolidate evidence, develop economic narratives, and drive policy reforms to highlight nursing's financial contributions. By integrating economic, business, and political perspectives into nursing leadership, research, and education, the profession can establish itself as an indispensable strategic asset essential to the sustainability of healthcare systems. Recognizing nursing's economic impact is a professional obligation and a vital step toward ensuring healthcare's long-term viability.
